# Microendoscopic Ultrasound-Guided Percutaneous Tracheostomy (MUGPT): A Case Series Describing a Novel Technique for Performing Percutaneous Tracheostomy

**DOI:** 10.1155/2023/5796473

**Published:** 2023-11-18

**Authors:** Mayyas M. Msheik, Amro F. Khalili, Mustapha A. Nahle, Chakib M. Ayoub, Yara M. Al Ghabour, Hachem Y. Abdul-Kader, Marwan S. Rizk

**Affiliations:** ^1^Department of Surgery, American University of Beirut Medical Center, Riad El-Solh, 1107 2020 Beirut, Lebanon; ^2^Department of Anaesthesiology, American University of Beirut Medical Center, Riad El-Solh, 1107 2020 Beirut, Lebanon; ^3^Department of Anaesthesiology, Rafic Hariri University Hospital, Bir-Hasan, Beirut, Lebanon; ^4^Department of Anaesthesiology Duke Health, 5673 HAFS, Durham, North Carolina, USA; ^5^Department of Anaesthesiology Lebanese University, Rafic Hariri University Campus, Hadath, Lebanon

## Abstract

A percutaneous tracheostomy is a common surgical procedure done in intensive care. Several different techniques have been described. Recently, the addition of bronchoscopy or ultrasound has been advocated to decrease the risks and complications associated with the procedure; however, both aids used alone, bronchoscopy or ultrasound, have some drawbacks and pitfalls. In this manuscript, we describe a new technique implementing a new technology, Microendoscopy coupled with ultrasound to perform percutaneous dilation tracheostomy MUGPT. MUGPT relies on dual real-time feedback microendoscopy and ultrasound to perform percutaneous dilation tracheostomy. This technique helps reduce the risk of bleeding, airway loss, tracheal wall injury, tracheal ring fracture, damage to adjacent structures, pneumothorax, pneumomediastinum, subcutaneous emphysema, false placement, hypoxia, carbon dioxide retention bronchospasm, cardiac dysrhythmias, and cost reduction. *Methods*. This is a case series of 6 patients who underwent single-step percutaneous dilation tracheostomy using the MUGPT technique. All the patients were in ICU and were candidates for tracheostomy. Intraoperative data collection, vital signs, oxygen saturation, and end-tidal CO2 were measured. No postoperative or intraoperative complications were documented. *Conclusion*. Microendoscopic ultrasound-guided percutaneous tracheostomy (MUGPT) is a promising technique with minimal complications. It is a procedure that can be performed and taught easily to Junior physicians and is a lifesaver in difficult cases.

## 1. Introduction

Percutaneous tracheostomy (PT), a well-established and common procedure performed in intensive care units, is still one of the riskiest procedures [1].

Several modifications to the procedure have been implemented since its inception in the Middle Ages. In 1969, Ciaglia introduced the wire-guided Seldinger technique, and a few years later, several improvements and modifications have been advocated by Griggs, and Fantoni, such as Percu Twist technique [1, 2]. Currently, the most common is a single-step percutaneous dilation tracheostomy [3], which can be ultrasound or bronchoscopy-guided [4–6]. The variation in attempts came out to improve and lower complications associated with PT and was addressed based on the patient selection in determining whether surgical or PT should be performed [7]. Microendoscopy ultrasound-guided dilation percutaneous tracheostomy (MUGPT) stands as progress in the approaches to performing PT, taking into account the drawbacks and challenges faced with previous techniques and reducing most.

Our case series consisting of six patients describes MUGPT, a new and safe technique for performing PT, relying on dual real-time images simultaneously displayed on one screen of both bronchoscopy and ultrasound. By applying this approach, we aim to reduce the risk of bleeding, airway loss, tracheal wall injury, tracheal ring fracture, damage to adjacent structures, pneumothorax, pneumomediastinum, subcutaneous emphysema, false placement, hypoxia, carbon dioxide retention bronchospasm, cardiac dysrhythmias, aspiration, conversion to an open surgical tracheostomy, and even death [1].

## 2. Methods and Ethics

The MUGPT uses a novel specialized device that has both an ultrasound and a microendoscope displayed on one screen and allows the real-time visualization of all steps.

Ethical approval was granted for this human clinical study by the Lebanese Army Ethical and Legal office, headed by Commander in Chief General Brigade Joseph Aoun, 1853/MC/MH, reference number 1/11310 Military Personnel Staff Office, Lebanese Military Army Head Quarters, Ministry of Defense Lebanon; Yarze February 19, 2022. All our patients were unconscious or sedated so informed consent was obtained from their legal guardians, who were informed about the MUGPT procedure and how it uses a new device to perform the percutaneous dilation tracheostomy, and written informed consent was obtained before each procedure.

### 2.1. Patient Information

Our case series included both male and female patients, with three of them being obese with short necks. Before performing the procedure, consent was obtained from the legal guardians and a detailed explanation of the risks and benefits of the procedure was provided to them. All patients were intubated for a minimum of two weeks before the tracheostomy was performed.

#### 2.1.1. Patient 1

A 78-year-old male with a medical history of hypertension, diabetes mellitus, anemia of chronic disease, and a previous cerebrovascular accident (CVA). He was admitted to the hospital for type 1 respiratory failure secondary to pneumonia. Due to his prolonged intubation and failure to extubate, a decision was made to perform a tracheostomy on 24/11/2021, roughly one month after his admission. The prolonged intubation was attributed to residual weakness secondary to his previous CVA. This patient was not obese and had proper neck length with ease in anatomical landmark identification.

#### 2.1.2. Patient 2

An 89-year-old female with a medical history of dyslipidemia and hypertension. She was admitted to the hospital for anoxic brain injury, postmyocardial infarction, and cardiac arrest. The patient was intubated upon admission on 30/11/2021, and a decision was made to perform a tracheostomy on 6/01/2022, due to the prolonged nature of her intubation. The patient was an obese individual with a short and thick neck; anatomical landmarks were not easily identified.

#### 2.1.3. Patient 3

An 80-year-old female with a medical history of hypertension, type 2 diabetes, and coronary artery disease. She was admitted to the hospital with pneumonia and subsequently intubated for type 1 respiratory failure. After two failed attempts to wean and extubate the patient, a decision was made to perform a tracheostomy on 15/12/2022. This patient had easily identified neck landmarks.

#### 2.1.4. Patient 4

A 65-year-old male with a medical history of congestive heart failure, hypertension, coronary artery disease, chronic obstructive pulmonary disease, type II diabetes, dyslipidemia, and hypothyroidism presented with a COPD exacerbation on 1/12/2022 and, during his hospital stay, suffered a cardiac arrest and hypoxic brain injury. Despite two attempts to extubate, the patient failed to wean, and the decision was made to perform a tracheostomy on 23/12/2022. The patient was an obese individual with a short and thick neck, and anatomical landmarks were not easily identified.

#### 2.1.5. Patient 5

A 75-year-old female with a history of hypertension and dyslipidemia presented with a myocardial infarction and subsequently suffered an anoxic brain injury following a cardiac arrest. The patient was admitted to the hospital and intubated on 12/11/2022. Due to prolonged mechanical ventilation and failure to wean, a decision was made to perform a tracheostomy. The procedure was performed on 26/01/2023. The patient had a proper neck distance and was not obese.

#### 2.1.6. Patient 6

A 70-year-old male with a history of hypertension presented postfall with a subdural hematoma on 15/12/2022. The patient was intubated on 27/12/2022 due to a decreased level of consciousness and subsequently failed to extubate twice, attributed to pneumonia, left vocal cord paresis, and right cord paralysis.

### 2.2. Intervention

MUGPT, a single port entry, is utilized to access the anterior wall of the trachea under dual ultrasound and visual guidance. The needle is inserted perpendicular to the trachea, between the second and the third tracheal ring, under ultrasound guidance, with the bevel facing down. The microendoscope in the needle allows the operator to have a double real-time view of the needle hub by ultrasonography and at its tip by endoscopy. This will safely guide the needle toward the tracheal lumen and precisely confirm its right position. After the withdrawal of the microendoscope, the operator will proceed with the Seldinger technique. The needle bevel is kept in this downward position to help direct the guidewire into the distal trachea. Over the guiding wire, a single-step dilation is done, and a tracheostomy tube is inserted. The position is confirmed by the insertion of the microendoscope inside the tracheostomy and visualization of the carina and inner tracheal lumen.

### 2.3. Technique Description

For all cases except patient 5, a second assistant (MD), residents, and a biomedical engineer from the ultrasound company were present during the procedure.

All the patients had normal INR and platelet counts above 100 K before the procedure.

All patients were attached to full vital sign monitors, endotracheal suctioning was performed, and a leak test was done with positive results on all patients. Afterward, patients were deeply sedated with fentanyl and midazolam and paralyzed with rocuronium.

Asepsis and antisepsis were performed from the mandibular angle to the second thoracic ribs. Full body draping was done with exposure of the area from the angle of the mandible, whole neck, and upper chest till the second rib.

Two approaches were used that did not affect the outcome but were done by the operator to check the visibility of the ET tube by microendoscopy, one where the endotracheal tube position was adjusted cephalad (A) and approach NA endotracheal tube position was not adjusted.

**Figure 1:**
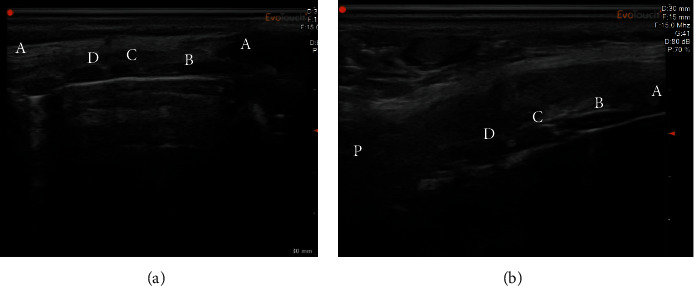
(a) Longitudinal ultrasound image of the neck: (A) cricoid cartilage, (B) 1st tracheal ring, (C) 2nd tracheal ring, and (D) 3rd tracheal ring. (A) Anterior side. (b) Longitudinal ultrasound image of the neck: (A) cricoid cartilage, (B) 1^st^ tracheal ring, (C) 2^nd^ tracheal ring, and (D) (3^rd^) tracheal ring. (P) Posterior side.

**Figure 2:**
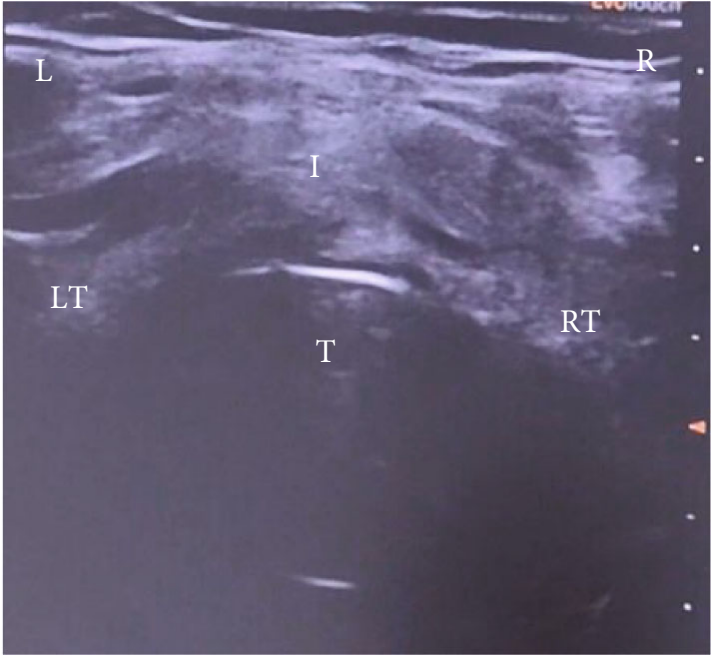
Cross-sectional view of the neck visualizing thyroid and tracheal anatomy. (T) trachea, (RT) right thyroid lobe, (LT) left thyroid lobe, (I) isthmus, (R) right, and (L) left.

**Figure 3:**
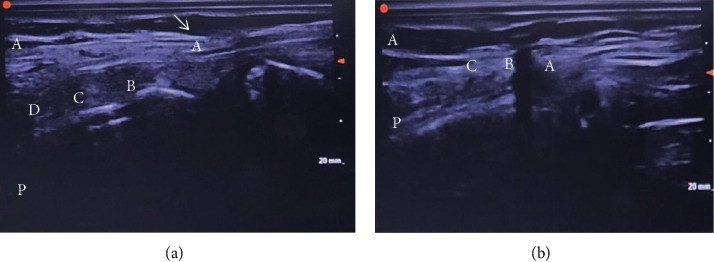
Needle localization on ultrasound longitudinal view. (a) (A) cricoid cartilage, (B) 1st tracheal ring, (C) 2nd tracheal ring, and (D) 3rd tracheal ring; (white arrow) needle black shadow. (A) Anterior side and (P) posterior side. (b) (A) cricoid cartilage, (B) 1^st^ tracheal ring, and (C) 2^nd^ tracheal ring; (white arrow) needle black shadow between (B) and (C). (A) Anterior side and (P) posterior side.

**Figure 4:**
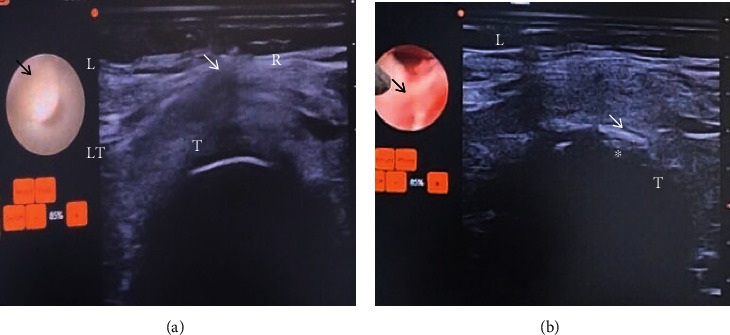
(a, b) Cross-sectional view of neck locating midline structures: thyroid trachea. (a) (T) trachea, (LT) left thyroid lobe, (white arrow) needle shadow, (black arrow) tracheal lumen, (R) right, and (L) left. (b) (T) trachea, (black arrow) tracheal lumen, (white arrow) needle shadow, (^∗^) anterior superior tracheal wall, (R) right, and (L) left.

**Figure 5:**
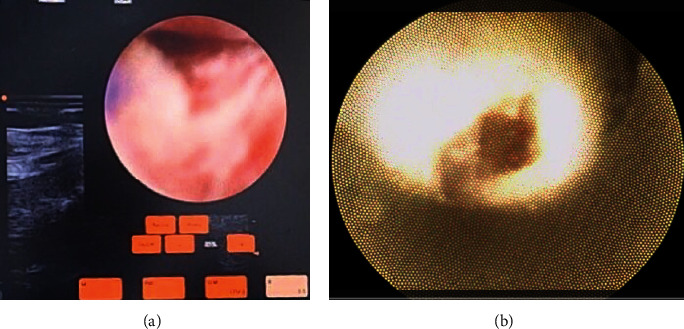
(a, b) Real-time microendoscope image showing intratracheal cartilage. (a) Intratracheal membrane tissue. (b) Hydrodissection with saline and anterior tracheal wall membrane between tracheal rings 2 and 3.

**Figure 6:**
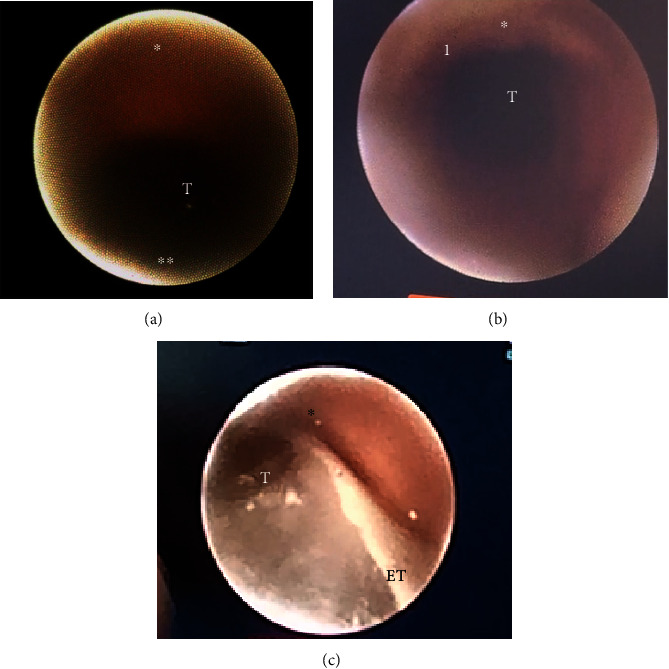
(a, b) Real-time view or the tracheal lumen: (T) Trachea lumen, (^∗^) anterior superior tracheal wall, (^∗∗^) posterior tracheal muscular wall, (1) tracheal rings. (c) Real-time scope image intratracheal cartilage dissection: (T) tracheal lumen, (^∗^) anterior superior tracheal wall, and (ET) endotracheal tube.

**Figure 7:**
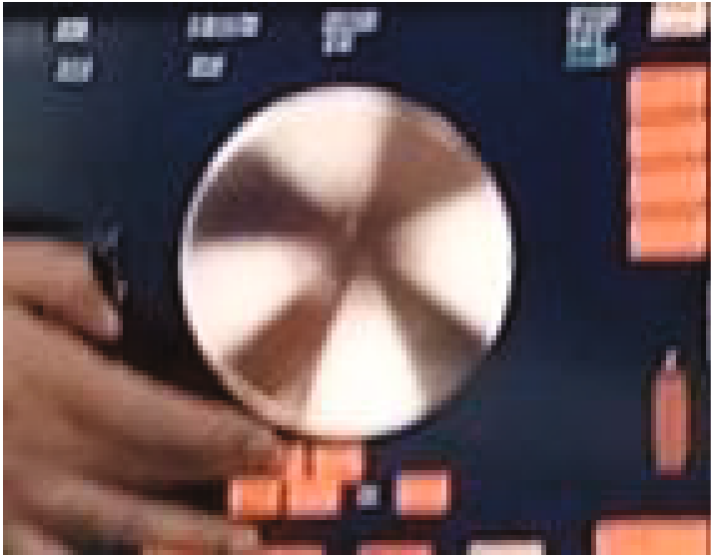
Real-time view within the Angio catheter.

**Figure 8:**
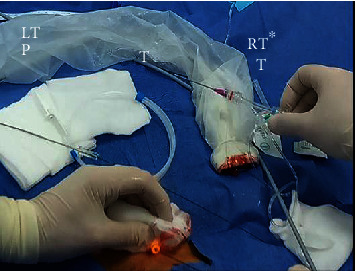
Wire placement and needle withdrawal.

**Figure 9:**
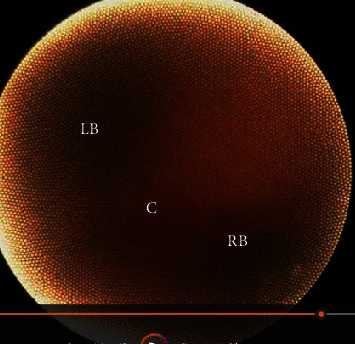
(C) carina, (LB) left bronchus, and (RB) right bronchus).

Procedure steps, ultrasound, and microendoscopic images:

1. Materials are checked and ready refer to Appendix A for the used needed items
2. The patient's neck is extended to expose the area where the tracheostomy will be performed
3. The neck and upper chest are sterilized to prevent infection during the procedure
4. Antibiotics are administered before the procedure to prevent infection
5. The patient is draped from the second rib up to the angle of the mandible with the whole neck and clavicular notch exposed to ensure a sterile field
6. The physician uses a linear phased array probe to scan the neck and localize the cricoid cartilage, tracheal rings 1st, 2nd, and 3rd, ET tube, thyroid gland, and superior thyroid artery and veins (Figures 1(a) and 1(b) and 2). The spaces are localized by longitudinal transection of the trachea starting cephalad at the cricoid and caudad till the 3rd tracheal ring and then between the 1st and 2nd tracheal rings or 2nd and 3rd, using a 22 gage needle slide under the ultrasound probe (Figures 3(a) and 3(b). The localized space is then marked
7. Local anesthetic with epinephrine is infiltrated at the marked space
8. An echogenic needle 14 Fr is inserted under ultrasound guidance to the 1 mm distance from the tracheal space 1st and 2nd/2nd and 3rd, (Figures 4(a) and 4(b)).
9. Hydrodissection with 2 to 3 ml of saline is performed to visualize the anterior tracheal wall by the camera (Figures 5(a) and 5(b)).
10. The space between the tracheal rings is pricked under vision using the scope present in the needle, and the entry into the trachea is made (Figures 6(a)–6(c)).
11. If ET position is not adjusted, the balloon and tube should be visualized (Figure 6(c)), or if the tube is withdrawn cephalad, then the tracheal ring will be visualized from the inside, and the posterior muscular wall of the trachea should be seen (Figures 6(a) and 6(b))
12. The 14 Fr soft angiocath is slid in, and the metallic part is withdrawn (Figure 7).
13. Recheck with fine scope to confirm the soft Angio catheter is in the tracheal and not touching the posterior wall and is directed caudad and anteriorly (Figure 7).
14. The scope is withdrawn, and the wire is inserted (Figure 8).
15. The 2 cm incision horizontally is done by a 13 blade. Serial dilation is performed to create a tract for tracheostomy tube insertion. The endotracheal tube is withdrawn at this step
16. After the tracheostomy tube has been inserted, the position needs to be confirmed by inserting a fine camera scope through the tracheostomy tube to visualize the carina and tracheal rings (Figure 9).
17. The procedure is then completed by connecting the tracheostomy tube to the ventilator and confirming adequate ventilation and oxygenation by end-tidal capnography

## 3. Discussion

Our case series is a description of how to implement the use of new technology microendoscopy coupled with ultrasound in performing percutaneous dilation tracheostomy and prove it a safe and feasible procedure, with minimal risk of bleed, no risk of ET tube balloon perforation, no risk of posterior tracheal wall puncture, and no events of hypoxia or carbon dioxide retention. In our case series, MUGPT was done with the same level of comfort in obese and nonobese patients.

Tables 1–4 show that during the whole time of our procedure, no hypoxia or increase in carbon dioxide was observed. Increased blood pressure was documented in two steps, one was at the administration of a local anesthetic and epinephrine infiltration, and the second was during the single-step dilation. Also, no major bleeding was reported intraoperatively or postoperatively. Moreover, the ventilation rate was kept the same during the procedure, and at no time did any changes in lung mechanics or airway pressures occur. In Table 5, an ample amount of time was taken to perform the procedure.

Our procedure, MUGPT, troubleshoots most of the difficulties and risks. First, there is no need for circuit disconnection and no need for an increase in endotracheal tube size to fit the bronchoscope, as our port of entry and visual feedback is through the same channel, which reduces exposure to aerosolized particles. Second, the risk of bleeding is minimal as ultrasound and visual feedback can provide unquestionable accuracy for the location of vessels during our procedure. Third, the risk of tracheal stenosis is also minimal, as only one puncture and one trial were needed to carry out this procedure, and the position of tracheostomy and site of entry can be guaranteed to be midline in the trachea and not bordering on any structure. Fourth, no hypoxia or carbon dioxide retention occurred even when the procedure lasted for more than twenty minutes. Finally, there is no change in lung pressures as the size of the cannula is very small (16 French) and does not occlude the airway or increase the risk of barotrauma, yet it can provide proper visual feedback of inner tracheal structures.

Percutaneous dilation tracheostomy is usually performed at the bedside in the intensive care unit. There are two approaches—anatomical or ultrasound-guided, both with the aid of endotracheal bronchoscopy [4–6]. The use of bronchoscopy guidance during PT can be helpful in preventing injury to adjacent structures, ensuring proper positioning of the tube, avoiding damage to the posterior tracheal wall, and confirming the correct placement within the tracheal lumen. However, this requires specialized expensive equipment in addition to a staff of specific skills. The use of flexible bronchoscopy carries several limitations, and the presence of blood in the trachea, even in small amounts, can significantly affect visibility [8, 9]. In addition, it is recommended to use a bronchoscope with an outer diameter (O.D.) of at least 2 mm less than the inner diameter (I.D.) of the endotracheal tube to avoid complete or partial tracheal occlusion leading to air trapping and overinflation by valve effect, increasing the risk of barotrauma [9]. Furthermore, deep neuromuscular blockade, ventilation with lower pressures, smaller tidal volumes, and higher respiratory rates or insertion of a double-lumen endotracheal tube for doing PT are recommended interventions to decrease the risk of barotrauma and pneumothorax [9–11].

Reports suggesting the use of real-time ultrasonography (US) guidance during PT can be particularly helpful when performing the procedure on patients with factors that make it technically challenging, such as morbid obesity, difficult anatomy, and cervical spine limited mobilization [12]. In these cases, ultrasound imaging can accurately determine the position of the tracheal rings before puncturing, even in patients where the tracheal anatomy cannot be clearly palpated due to morbid obesity or when the neck cannot be extended due to cervical spine abnormalities [12]. A randomized controlled trial on thirteen patients, using US-guided PT, showed that the latter is reproducible, accurate, and safe, even for patients with morbid obesity. No hindrances or complications were detected during the study [12]. US guidance also reduces operation time compared with bronchoscopy guidance [13].

Patients with respiratory failure and low pulmonary reserve, even 150 to 200 mL of blood in the trachea, can lead to severe hypoxia and an inability to ventilate [8]. Therefore, intraoperative tracheal hemorrhage can be life-threatening, even with low blood loss. US guidance helps in puncturing the trachea above the third tracheal ring, detecting abnormal vascular anatomy, and identifying major superficial veins to avoid injury to these vessels, 2% to 12% of the population have a low thyroid artery, so the use of ultrasound avoids fatal hemorrhagic incident [10, 11, 14, 15].

The number of attempts and puncture sites and the improper location of the tracheostomy tube increase the risk of tracheal stenosis. Consequently, it is important to determine, with complete accuracy, the location of the puncture site to be the midline position of the trachea [16–19].

Minimizing exposure to aerosolized particles while performing a percutaneous dilation tracheostomy should be achieved by maintaining a closed circuit and a small port of entry. Consider doing a percutaneous dilation tracheostomy in patients with the coronavirus disease (COVID-19); the operator, in addition to the use of full personal protective equipment (PPE), needs a closed ventilatory circuit with deep neuromuscular blockade to reduce the cough reflex and results in a reduction of exposure to virulent particles.

To conclude, MUGPT is a promising and safe technique with potential demonstrated through our case series. The importance of our technique stands out for the following facts. First, MUGPT can be done very slowly with utmost accuracy; patients are not at risk of desaturation, carbon dioxide retention, or lung injury due to barotrauma, and staff are not at increased risk of infection. Why is this important? The key elements for a successful procedure are two: one is patient safety and satisfaction, and the second is the ability to use it for teaching with reproducible outcomes; this was achieved by our method. Second, MUGPT gives a good exposure to properly correlate microendoscopic anatomical images with ultrasound images of anatomical structures. Finally, MUGPT is a lifesaver in difficult-to-ventilate and intubate cases, as even an inexperienced anesthesiologist can create a safe surgical airway with complete accuracy and may not need help from seniors or ENT specialists.

In conclusion, we must acknowledge that our study had some limitations. First, our sample size was small 6 patients. Second, MUGPT should be compared to other techniques, bronchoscope, or ultrasound alone, in the format of a large randomized controlled trial. Third, all the procedures were carried out by the same physician who is well-experienced in ultrasound and microendoscopy. Our message, first, is to show the functionality of MUGPT as a new approach for performing percutaneous dilation tracheostomy by implementing new technology for the benefit of the patient. Second, providing new solutions and emphasizing the benefit of new technology as a saver when urgent lifesaving procedures of establishing an artificial airway are a challenge to experts. Third, most ICUs worldwide rely significantly on ultrasound in medical management and perform quite a few procedures; why not add a new technology and couple it to our current knowledge to perform one of the riskiest procedures in intensive care?

Our aim is for MUGPT to be a reproducible and easy technique and used as a teaching modality for residents, from all fields of specialty, who need to handle airway emergency medicine, anesthesia, surgery, critical care, and ENT.

## Figures and Tables

**Table 1:** Blood pressure time at zero minutes (start of procedure), 5, 10, 15, 20 and 30 minutes.

Case	BP T0	BP T5	BP T10	BP T15	BP T20	BP T30
Patient 1	143/78	162/87	140/76	153/83	150/76	144/76
Patient 2	130/64	155/76	150/74	150/78	147/76	135/62
Patient 3	140/61	167/82	150/72	160/77	143/65	140/63
Patient 4	137/67	148/78	130/60	156/73	152/74	134/65
Patient 5	124/54	155/82	144/71	120/63	110/60	120/57
Patient 6	150/84	165/90	155/84	155/85	157/84	155/82

BP: blood pressure; T: time.

**Table 2:** Heart rate during the procedure.

Case	HR T0	HR T5	HR T10	HR T15	HR T20	HR 30
Patient 1	80	90	88	96	94	87
Patient 2	70	84	83	105	103	92
Patient 3	86	110	100	94	92	82
Patient 4	74	87	83	90	94	90
Patient 5	65	93	75	73	66	60
Patient 6	75	85	80	82	90	80

HR: heart rate.

**Table 3:** SpO2 on FiO2 100 during the procedure.

Case	SpO2 T0	SpO2 T5	SpO2 T10	SpO2 T20		
Patient 1	98	98	98	98	98	98
Patient 2	100	100	100	100	100	100
Patient 3	99	99	99	99	99	99
Patient 4	99	99	99	99	99	99
Patient 5	100	100	100	100	100	100
Patient 6	98	99	99	99	99	99

SpO2: oxygen saturation; FiO2: fraction of inspired oxygen.

**Table 4:** EtCO_2_ during procedure.

Case	EtCO_2_ T0	EtCO_2_ T5	EtCO_2_ T10	EtCO_2_ T15	EtCO_2_ T20	EtCO_2_ T30
Patient 1	40	42	40	41	40	38
Patient 2	30	33	35	35	36	35
Patient 3	38	41	38	38	33	33
Patient 4	33	35	34	38	38	37
Patient 5	36	36	34	34	34	34
Patient 6	NA	NA	NA	NA	NA	NA

EtCO_2_: end tidal CO_2_.

**Table 5:** Operative timing.

Case	Procedure time (not including sterilization and draping/actual work from scanning to tracheostomy)	Procedure time with draping and prior scanning
Patient 1	30 min	80 mins
Patient 2	18 mins	45 mins
Patient 3	13 mins	40 mins
Patient 4	18 mins	40 mins
Patient 5	5 mins	25 mins
Patient 6	40 mins	90 mins

Mins: minutes.

## Data Availability

Tables and images presented in this article are not present elsewhere and are property of the first author; however, these figures and tables can be found in the supplemental files under figure and tables.

## Appendix

### Equipment, Items, and Medication

- Ultrasound with microendoscope (Quantel and 7star scope) description of machine
- Percutaneous tracheostomy kit
- Monitor
- Ventilator
- Syringes 3 × 10 ml, 2 × 20 ml
- Needles 25 gage, 22, 20, and 18
- Special 3-way device handling kit (port for scope, port for wire, and flushing-suctioning port)
- Local anesthetic with epinephrine
- Sedative (fentanyl and midazolam)
- Neuromuscular blocker
- Suction catheter
- Ky gel
- Universal drape
- Camera cover
- Sterile surgical gowns 2x
- Sterile gloves 4x
- Laryngoscope
- ET tubes 6, 7, and 8
- Ambu bag
- Marking pen
